# Detection of Unlabeled Polystyrene Micro- and Nanoplastics in Mammalian Tissue by Optical Photothermal Infrared Spectroscopy

**DOI:** 10.1021/acs.analchem.4c05400

**Published:** 2025-08-01

**Authors:** Kristina Duswald, Verena Pichler, Verena Kopatz, Tanja Limberger, Verena Karl, David Hennerbichler, Robert Zimmerleiter, Wolfgang Wadsak, Mike Hettich, Elisabeth S. Gruber, Lukas Kenner, Markus Brandstetter

**Affiliations:** † RECENDT GmbH—Research Center for Non-Destructive Testing, Linz, Upper Austria 4040, Austria; ‡ CBmed GmbH—Center for Biomarker Research in Medicine, Graz, Styria 8010, Austria; § Department of Pharmaceutical Sciences, Division of Pharmaceutical Chemistry, University Vienna, Vienna 1090, Austria; ∥ Clinical Institute of Pathology, Department for Experimental and Laboratory Animal Pathology, Medical University of Vienna, Vienna 1090, Austria; ⊥ Comprehensive Cancer Center, Medical University Vienna, Vienna 1090, Austria; # Department for Radiation Oncology, 27271 Medical University of Vienna, Vienna 1090, Austria; ∇ Unit of Laboratory Animal Pathology, University of Veterinary Medicine Vienna, Vienna 1210, Austria; ○ Department of Molecular Biology, Umeå University, Umeå 90187, Sweden; ◆ Christian Doppler Laboratory for Applied Metabolomics, Medical University Vienna, Vienna 1090, Austria; ● Department of General Surgery, Medical University Vienna, Vienna 1090, Austria

## Abstract

In this study, we investigate the efficacy of optical photothermal infrared (O-PTIR) spectroscopy, also known as mid-infrared photothermal (MIP) microscopy, for label-free and nondestructive detection of micro- and nanoplastics (MNPs) down to diameters of 200 nm in mammalian tissues. Experiments with both *in vitro* three-dimensional cell cultures derived from HTC116 colorectal cancer cell line and *in vivo* mouse tissue models were conducted. Spherical polystyrene particles served as reliable model systems for evaluating spatial resolution limits and quality of spectra. Our findings demonstrate the superior resolution of O-PTIR in imaging individual particles of 200 nm in mouse kidney tissues, surpassing the capabilities of traditional Fourier transform infrared (FTIR) spectroscopy. Furthermore, we apply a semiautomated image analysis that incorporates machine learning algorithms to accelerate the detection process, thus improving throughput and minimizing the potential for human error. The results confirm that O-PTIR is able to provide high-quality, artifact-free spectral images in a contact-less manner and significantly outperforms traditional infrared spectroscopy in terms of spatial resolution and signal-to-noise ratio in complex biological matrices.

## Graphical abstract

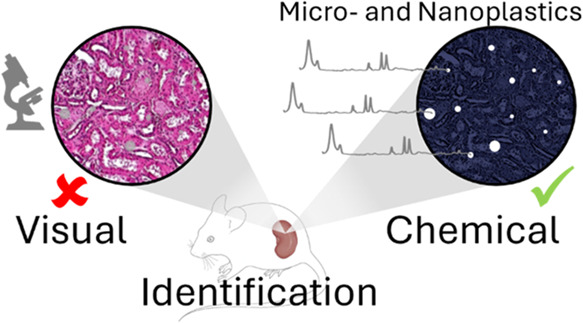

Microplastics are an increasing concern in daily life, absorbed by living organisms and found globally—on land, in the air, in bodies of water,^1–3^ and even in remote places such as Arctic areas,^4^ isolated regions of the Pyrenees Mountains,^5^ or Arctic beaches on remote islands.^6^ The full extent of the consequences remains largely unknown.^7,8^ A WHO^9^ study recently summarized research on human exposure to micro- and nanoplastic (MNP) particles, highlighting the need for reliable detection methods for particles smaller than 10 μm. Particles in that size range can easily be taken up, primarily via ingestion,^10,11^ but also through inhalation^12^ or by breaching the cutaneous barrier,^13^ leading to accumulation in the tissue.^14^

Recent studies suggest that frequent exposure to high levels of microplastics may increase the risk of diseases like colorectal cancer (CRC).^15,16^ Several studies have suggested that microplastics promote metastasis in CRC^17^ and breast cancer cells.^18^ These particles can remain in the cells and may even be passed on during cell division. It has recently been shown that cholesterol molecules facilitate MNPs in breaching the blood-brain barrier in mice.^19^ Kopatz^19^ and Kaushik^20^ have highlighted the threat posed by MNPs, which can cross the blood-brain barrier and potentially cause severe neurotoxicity. By conducting label-free determination of cancerous and noncancerous tissues in human bladder surgical specimens using Raman spectroscopy, Krafft^21^ demonstrated the utility of this technology for intraoperative tissue detection and simultaneously identified the presence of microplastics, pigments, and other foreign materials within the tissue samples. Since MNPs are abundantly found in human tissues, there is a pressing need to develop dedicated detection methods that accurately assess their presence and impact. However, current analytical techniques for MNP detection and their interactions with living organisms are still nascent.^8^ Thus, fast and reliable methods for localization and identification of MNPs in biological samples are prerequisites for better understanding their impact on our health. Various studies have suggested methods to address these questions, at least partially, such as pyrolysis-based techniques,^22^ microscopic techniques,^23,24^ and vibrational spectroscopy. Concerning the latter, Fourier-Transform Infrared Spectroscopy (FTIR)^25,26^ and Raman spectroscopy^27^ have become reliable tools for identifying microplastics based on their unique chemical fingerprint. However, each method has its respective strengths and weaknesses. FTIR is usually only applied to detect particles with sizes above 10 μm due to a limited spatial resolution imposed by the optical diffraction limit and can also suffer from artifacts in reflection measurements that are difficult to interpret. Conversely, Raman spectroscopy can detect particles below diameters of 10 μm unless the sample exhibits fluorescence, which can superpose the polymer characteristics in the spectrum.^27^ Attenuated Total Reflection (ATR), a robust sampling mode of FTIR, is less prone to artifacts and unaffected by fluorescence, unlike other FTIR modes and Raman techniques. However, the ATR crystal must be in direct contact with the sample surface.^28^ Therefore, this method has a high potential for causing cross-contamination and lacks the necessary spatial resolution. These issues have stimulated the development of Optical Photothermal Infrared Spectroscopy (O-PTIR). The O-PTIR method combines IR spectroscopy principles with photothermal detection, where a sample absorbs a laser pulse, and the subsequent localized heating and accompanying expansion in the material is measured using visible light optics.^29^ This allows for the collection of IR spectra beyond the IR-diffraction limit, thus enabling detailed chemical analysis at the submicron scale without requiring direct contact with the sample.^30,31^

Research on microplastics detection in tissue has mainly focused on isolating polymer particles from biological material. To do this, the surrounding biological tissue is usually removed, making the particles more straightforward to analyze. Different chemicals can be used to dissolve the tissue surrounding the microplastics.^32–34^ However, this process is time-consuming and carries the risk of changing the chemical constitution of microplastics or even unintentional removal of particular polymer types, which might lead to false measurement results.^35^ Furthermore, this process results in the loss of crucial spatial information, such as the exact location of the microplastics within a tissue. Knowing where and in which cells microplastics accumulate in the tissue is crucial as it provides insights into how microplastics might affect or change the tissue and, consequently, to assess their potential impact on human health and diseases.

This study demonstrates that MNPs with diameters as small as 250 nm can be detected precisely in mouse kidney tissue using the O-PTIR technology. This method allows for the detection of MNPs without isolating them, surpassing the current detection limits of similar techniques. This advancement closes the gap between high-resolution, time-intensive methods, such as electron microscopy, and faster optical spectroscopy techniques, thereby enhancing the applicability of reliable, position-sensitive MNP detection in tissues.

We present a comparative analysis of the absorption spectra obtained from FTIR and O-PTIR using polystyrene (PS) particles of defined and varied sizes. Subsequently, we demonstrate the detection of MNP particles within a controlled three-dimensional cell culture model (spheroids). This model helps to isolate potential influencing factors within a complex cell matrix akin to mammalian tissue. The cells are cultivated in a controlled environment, avoiding the generation of different functional tissues. This methodology is supported by the initial steps toward semiautomated data evaluation using a region-growing image segmentation algorithm.

Finally, we illustrate the feasibility of detecting MNP particles in actual mouse tissue. We emphasize how the high lateral resolution, surpassing the diffraction limit for IR wavelengths, enables the detection of MNP particles in a spheroid and actual mouse tissue down to a size of 250 nm in diameter. This highlights the method’s potential for detailed environmental and biological analyses with straightforward and rapid two-wavelength scans.

## Materials and Methods

### Micro- and Nanoplastic Particles

Commercially available unlabeled and labeled PS particles with a diameter of 10.39 ± 0.13 μm (spherical, aqueous suspension, 5% w/v, blue colored), 1.14 ± 0.03 μm (spherical, aqueous suspension at 2.5% w/v, ex/em = 530/607 nm), 1.16 ± 0.04 μm (spherical, aqueous suspension at 5% w/v, red colored), 0.24 ± 0.01 μm (spherical, aqueous suspension at 2.5% w/v, ex/em = 502/518 nm) and 0.200 ± 0.007 μm (spherical, aqueous suspension, 5% w/v, unlabeled) were obtained from Microparticles GmbH (Berlin, Germany). As published, all particles were characterized by measuring zetapotential, size, and polydispersity index (PDI) on a Zetasizer Pro device (Malvern Pananalytical, Malvern, United Kingdom).^17^ We will refer to the particle sizes in the following as 10, 1 μm, 250 and 200 nm.

### HCT116 Spheroids

Human colorectal cancer cell line HCT116 (DSMZ, Braunschweig, Germany) was cultivated in a fully supplemented Minimum Essential Medium (MEM) culture medium (10% FBS, 1% P/S, 1% l-glutamine) at 37 °C and 5% CO_2_ in a humidified atmosphere. If not indicated otherwise, all cell culture reagents were bought from Sigma-Aldrich (St. Louis, Missouri, USA) and used as received. For spheroid formation, HCT116 cells were suspended with the respective MNPs (MNPs as described before, final MNP concentration 1 μg mL^–1^) in fully supplemented MEM media and seeded on Petri dish lids using the hanging drop method^36^ at concentrations of 3 × 10^3^ cells per drop. The MNP-cell suspension was required to ensure equal distribution of the MNPs within the spheroid. Other methods, like ultralow attachment plates or directly using agarose-coated plates, were unsuccessful, as the direct contact of the MNPs to the plastic surface of the cell culture plate led to uneven distribution within the spheroid. After 24 h, the medium was replaced by 10 μL of fresh media, and spheroids were transferred to agarose-coated 96-well plates with 100 μL of fresh media per well. MNP-treated spheroids (MNP concentration 1 μg mL^–1^) were embedded in Tissue-Tek O.C.T. Compound (Sakura, Torrance, California, USA) on day 7, and 7.5 μm thick cryosections were prepared on Superfrost microscope slides (Epredia, Kalamazoo, Michigan, USA) using a Leica CM3050 S cryomicrotome (Leica, Wetzlar, Germany) and stored at −80 °C. The samples were fixed two times with 4% paraformaldehyde (PFA) in phosphate-buffered saline (PBS) for 15 min and washed with PBS three times; afterward, the cells were dehydrated by an ethanol row applying 50, 70, 80, 95% and 3× 100%, each for 10 min.

### Preparation of *Ex Vivo* Samples

Kidneys from C57Bl/6J wildtype mice were extracted and post-mortem injected with 50 μL of PS-particle mix (0.3% w/v 10 μm blue colored particles, 0.03% w/v 1 μm red-colored particles and 0.015% w/v 200 nm unlabeled particles in PBS). The organs were then fixed for 24 h with 4.5% PFA before the samples were further desiccated and paraffin-embedded with a modified isopropanol protocol.^37^ Sections of 5 μm were cut and mounted on regular glass slides (SuperFrost Plus Adhesion Microscope Slides; Epredia, Breda, Netherlands). For removal of paraffin before analysis, samples were alternately heated up to 65 °C and quickly dipped into 100% isopropanol.^37^ The process was repeated several times until the tissue sections appeared uniformly whitish, and the surrounding paraffin was removed.

### Fourier-Transform Infrared (FTIR) Spectroscopy

FTIR is the gold standard in infrared spectroscopy to investigate the molecular composition and structure (“chemical fingerprint”) of the sample of interest. Each infrared absorption band corresponds to characteristic molecular vibrations, thus giving both qualitative and quantitative information about the specific functional chemical group.

Reference spectra were obtained with a Bruker LUMOS I FT-IR microscope (Bruker, Billerica, Massachusetts, USA), a fully automated IR device with a high-sensitivity, liquid nitrogen-cooled MCT detector. It enables transmission, reflection, and ATR mode, whereas the latter two have been used in this work. The microscope features an 8× objective, digital zoom up to 32×, a CCD camera, and a motorized sample stage for precise operation. The measurement spot size was controlled by adjusting the aperture. For the analysis of PS beads in reflection mode, a 10 × 10 μm^2^ aperture was used, and an increased 30 × 30 μm^2^ aperture was applied for all imaging applications to ensure sufficient signal-to-noise ratio (SNR).

The ATR system uses a motorized single-bounce ATR germanium crystal, which is lowered onto the sample to interact with the sample surface. The infrared light reflects at the interface, creating an evanescent field that extends a few micrometers into the sample. This method provides high-quality, robust and undistorted spectra, making it ideal for determining IR absorption bands. To obtain an optimum SNR, a 100 × 100 μm^2^ aperture was used for ATR measurements.

All spectra from the FTIR-microscope were obtained by conducting 32 scans for both the background and sample measurements. These were averaged to calculate the final IR absorption spectrum using Beer–Lambert’s law, *A* = −log_10_(*I*_s_/*I*_ref_), where *I*_s_ is a single beam reflection spectrum from the sample (particles) and *I*_ref_ is a reference or background single beam spectrum of a nonabsorbing material (e.g., air or gold mirror). The spectral range of all spectra obtained with the FTIR covers the wavenumbers between 4000 cm^–1^ and 600 cm^–1^.

### Optical Photothermal Infrared Spectroscopy

The mIRage system (Photothermal Spectroscopy Corp., Santa Barbara, California, USA) was used to acquire Optical Photothermal Infrared (O-PTIR) spectra through a colinear pump–probe approach. A tunable pulsed external-cavity quantum cascade laser, operating in the IR region from 3001 to 2677 cm^–1^ and 1853 to 933 cm^–1^, causes localized heating and thermal expansion in the sample. This photothermal effect is detected by a 532 nm CW-laser, with signal amplitude proportional to absorbed IR, enabling quantitative absorption spectra. A lock-in detection modulation scheme improves SNR.

Typical optical spot sizes are 500 nm (1/*e* ^2^) for the detection laser and 6.5–12 μm for the IR laser. O-PTIR spectra shown are averages of three scans. Additionally, the tunable laser was used to generate single-wavelength images, and the O-PTIR ratio image was calculated using intensities at 1455 and 1660 cm^–1^. The band at 1660 cm^–1^ corresponds to the Amide I band, primarily resulting from the C=O stretching vibration of peptide bonds in biological samples, while the band at 1455 cm^–1^ originates from methylene vibrations in the PS beads. This ratio highlights PS particles within tissue and normalizes intensity variations for accurate detection. While Raman spectroscopy could be done on the same spot, further experiments were abandoned due to long integration times and fluorescence issues.

To create a hyperspectral image, the investigated sample is scanned using a high-precision motorized XY-stage with a minimal step size of 100 nm. The step-size that was used for acquisition of the images shown herein is indicated in the respective figure caption.

### Image Processing

Two different types of images were obtained and used for image processing: single-wavelength images for particles larger than 200 nm, and hyperspectral images for detecting 200 nm particles in mouse kidneys. The applied region-growing algorithm^38,39^ is a segmentation technique that begins with seed points and expands to neighboring pixels based on predefined criteria, such as intensity similarity or connectivity. This approach iteratively includes adjacent pixels with properties similar to the seed point, forming a continuous region until no additional pixels meet the criteria.

To refine the segmentation results, postprocessing techniques, including morphological opening and closing, were applied. Morphological opening, which combines erosion followed by dilation, removes small noise artifacts and disconnects narrow connections. Morphological closing, consisting of dilation followed by erosion, fills small holes and smooths the edges of segmented regions.^40^ These processes were guided by a rectangular kernel, which defined the neighborhood structure and influenced the scale and orientation of features affected during refinement. The combination of these techniques ensured accurate segmentation with minimal artifacts and well-defined region boundaries.

Hyperspectral images were processed and analyzed by extracting spectral descriptors (ImageLab, Epina GmbH, Austria). Spectral descriptors are features that capture chemical composition and physical properties. These descriptors enable detailed analysis to identify the presence or absence of specific bands characteristic for PS, allowing particles to be precisely located at their exact positions.^25^

## Results and Discussion

We first compare the characteristics of O-PTIR with FTIR, the gold standard in IR spectroscopy, for the MNP beads investigated. We then continue to the main part of this paper, starting with experiments on MNPs incorporated into spheroids, thereby increasing sample complexity while maintaining a well-defined and controlled environment. Finally, we examine MNPs in tissue from mouse kidneys to demonstrate the method’s *in vivo* capabilities.

For a systematic investigation experiments were performed with four types of polystyrene (PS) beads of spherical shape, with average diameters of 10 μm (blue label), 1 μm (fluorescence or red label), 250 nm (fluorescence label), and 200 nm (unlabeled). Details on the MNPs and sample preparation are described in the Materials and Methods section above.

### FTIR and O-PTIR Performance on PS-Beads

In this section, O-PTIR results are compared to conventional FTIR microspectroscopic measurements in reflection and ATR geometry. Particles were placed on calcium fluoride slides to ensure interference-free reference spectra of PS in FTIR reflection measurements as well. A schematic of the size relations between FTIR (red) and O-PTIR (green) detection spots and the measured MNP particles is shown in Figure 1a, illustrating the enhanced spatial resolution of the O-PTIR technique. Although the diffraction limit of the pump beam is fundamentally the same as in the classical FTIR microscope, the diffraction limit of the probe laser and thus the diffraction limit of the whole system is much smaller (∼500 nm 1/*e* ^2^). The FTIR reflection (red line), and O-PTIR (green line) spectra for each particle size are presented in Figure 1b and labeled accordingly. The IR absorption spectrum taken in ATR measurement geometry is shown for reference as blue line at the top (standard Bruker OPUS ATR correction applied). Additionally, we applied Raman spectroscopy for MNP detection; however, strong autofluorescence in the mouse tissue prevented any meaningful results. All shown spectra are averaged from three subsequent measurements, with measurement times of 60 s (FTIR reflection), 60 s (FTIR-ATR), and 12 s (O-PTIR). It is clearly visible that FTIR measurements taken in reflection geometry are prone to artifacts, mainly caused by optical scattering effects,^41^ baseline and band shifts,^42^ and particle geometry, while the spectra obtained with O-PTIR are significantly less affected. The characteristic spectral fingerprint of the PS beads is clearly visible with a superior SNR even at a shorter measurement time of the O-PTIR system. To corroborate the recorded spectral information, a test measurement with an FTIR-ATR was conducted on the beads with an average diameter of 10 μm. The aperture size for FTIR reflection was 10 × 10 μm^2^, which matches the size of the 10 μm particles and is the smallest aperture that results in a viable SNR. For the FTIR-ATR measurements, an aperture of 100 × 100 μm^2^ was applied to obtain a reference spectrum with high quality.

**Figure 1:**
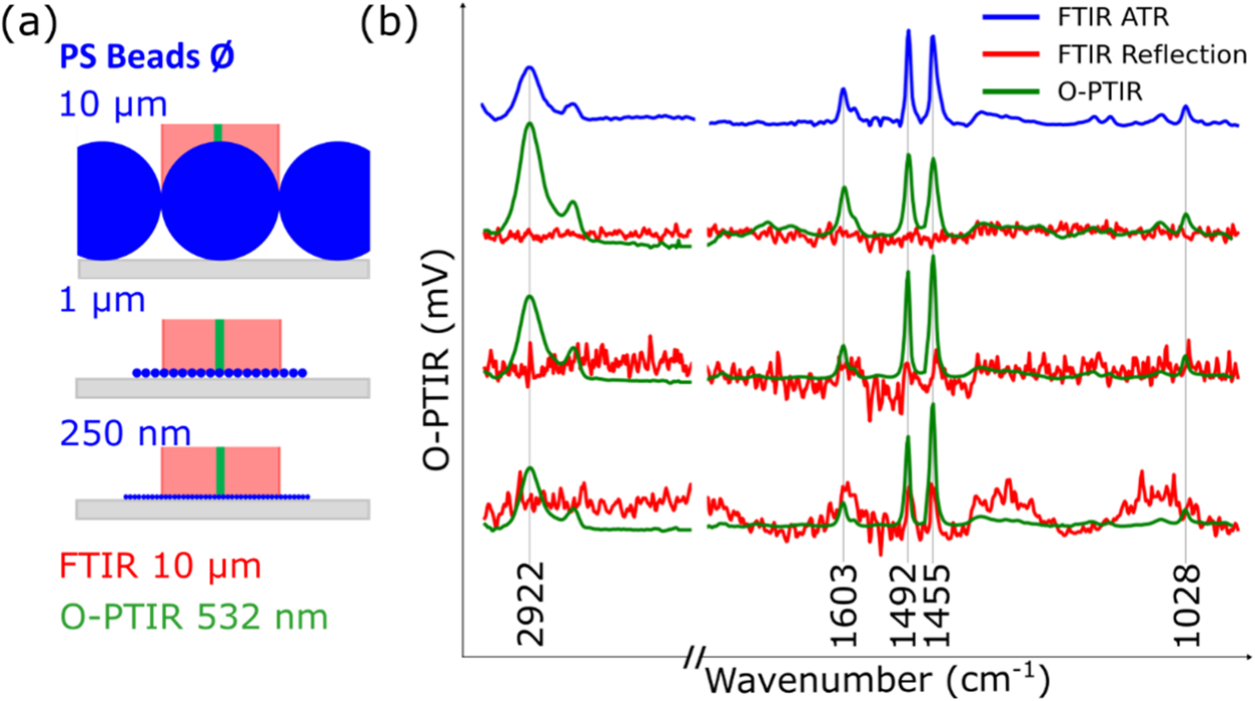
Comparison of the IR absorption spectra taken with three different methods—FTIR reflection (red), O-PTIR (green), and a reference FTIR-ATR (blue) for three different PS particle sizes (10, 1 μm, 250 nm). In (a), we show a schematic illustration of the effective lateral resolution: 10 μm for reflection FTIR (red) and 500 nm O-PTIR (green). Part (b) shows the FTIR-ATR reference spectra taken from 1 μm beads followed by the comparison of reflection FTIR and O-PTIR. The green top spectra were taken from 10 μm particles, the middle from 1 μm particles, and the bottom from 250 nm particles.

A comparison of the obtained spectra shows an excellent agreement between the O-PTIR and FTIR-ATR results. In contrast, FTIR reflection spectra barely contain usable information due to an insufficient SNR. In classical mid-IR microspectroscopy using thermal light sources, the use of apertures limits the achievable SNR, creating a trade-off between spatial resolution, SNR, spot size, and acquisition time. This trade-off becomes particularly challenging in scenarios with limited signal intensity, such as reflection measurements of poorly reflecting samples, as in biomedical diagnostic applications.^43^ Additional limiting factors are Rayleigh and Mie-scattering and the geometric scattering effects depending on the employed detection wavelengths. These effects have been quantitatively analyzed elsewhere^41^ and are also responsible for the decreasing PS absorption features with increasing particle diameter in the FTIR reflection spectra shown in Figure 1b. Although the O-PTIR and the FTIR-ATR methods are comparable in terms of their spectroscopic performance and SNR, the ATR method inherently demands that the crystal is in direct contact with the sample, which introduces the risk of cross-contamination and could cause alterations in the investigated samples due to the mechanical impact. Therefore, we used FTIR-ATR only as a complementary method for reference measurements in the context of MNP detection. Nevertheless, techniques like micro-ATR can in principle be used for micrometer-scale analysis.^44^

It is clear from the data shown in Figure 1b, that the O-PTIR system provides the necessary resolution for detecting nanoplastic particles (NMPs) in tissue. In contrast, the traditional setup suffers from low light intensity, making it difficult to obtain satisfying SNR levels, even with a 20 times larger measurement spot.

### MNP (PS-Beads) in HCT116 Spheroids

Based on the results presented in the previous section, MNPs with 1 μm and 250 nm diameters were incorporated within spheroids and measured using O-PTIR spectroscopy. This approach provided the basis for disentangling the cells and MNPs spectral fingerprints before proceeding to MNPs in mammalian tissue. Figure 2a compares an image from optical white-light microscopy to the corresponding O-PTIR ratio, which is smoothed using bilinear interpolation. The regions indicated by a white rectangle are exemplarily investigated in more detail in Figure 2b. The color bar represents the ratio of the absorption signals, as explained in the methods section. Thus, the red color indicates the occurrence of PS particles. The spots that appear in red indicate the presence of one or several PS beads (white numbers). Figure 3 shows spectra of individual PS beads (red), numbered according to their positions indicated in Figure 2b. It also includes spectra from pristine spheroids and PS MNPs embedded in a spheroid. Additionally, the figure highlights the wavenumbers specific to PS.

**Figure 2:**
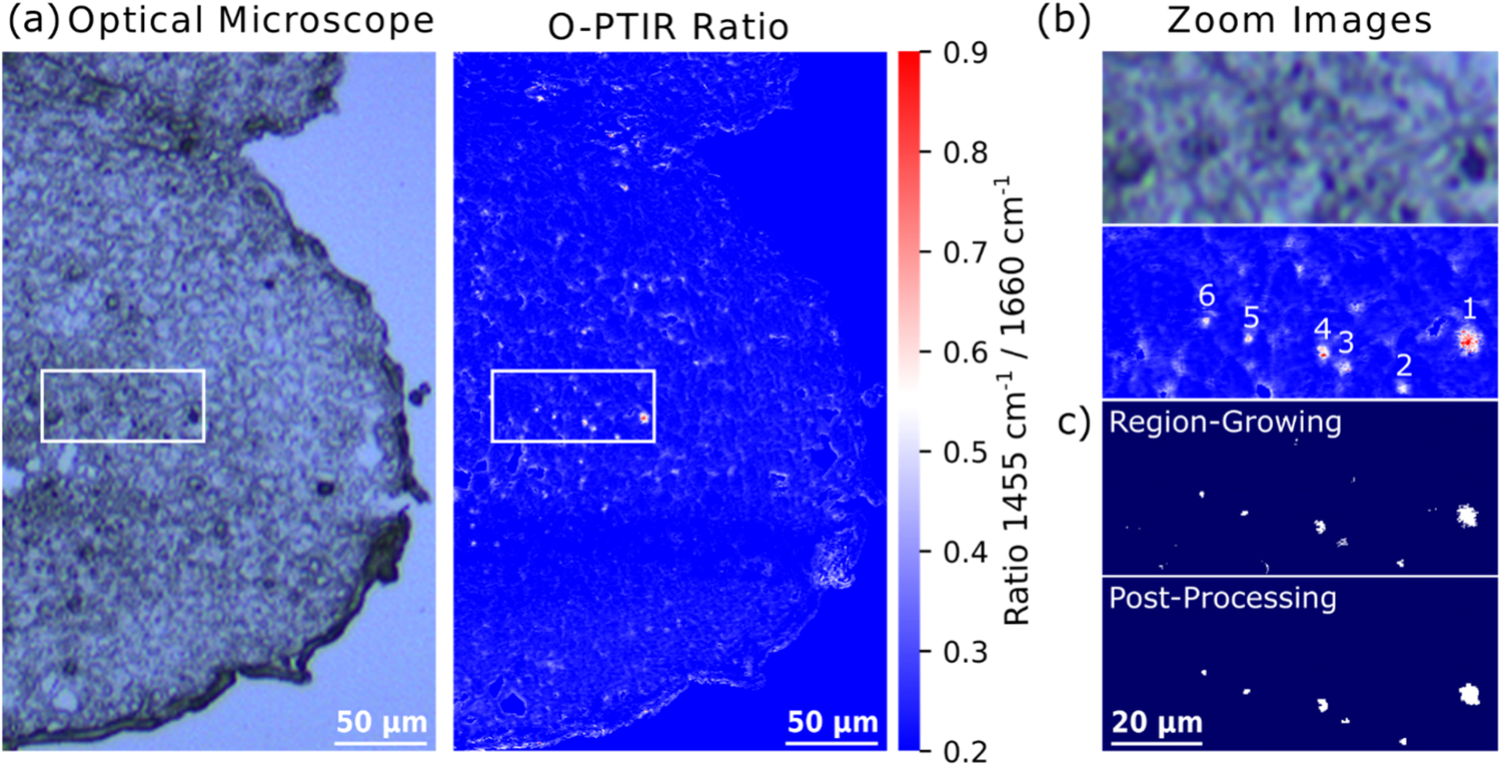
HCT116 spheroid was grown in fully supplemented MEM medium spiked with 1 μm PS spheres (1 μg mL^–1^). In (a) the left half shows a microscope image and the right half a false-color image of the same area where the color is given by the ratio of the absorbance, as explained in the methods section. Red shows a high presence of the characteristic bands of PS. (b) For better visibility the region marked by a white rectangle in (a) was analyzed in detail. A microscope image (top) and a false-color image (bottom) are compared, with the latter clearly showing the presence of PS-MNPs. (c) shows the segmentation of the ratio image after applying the region-growing algorithm (top). This segmentation was then postprocessed (bottom) to assign individual pixels.

**Figure 3:**
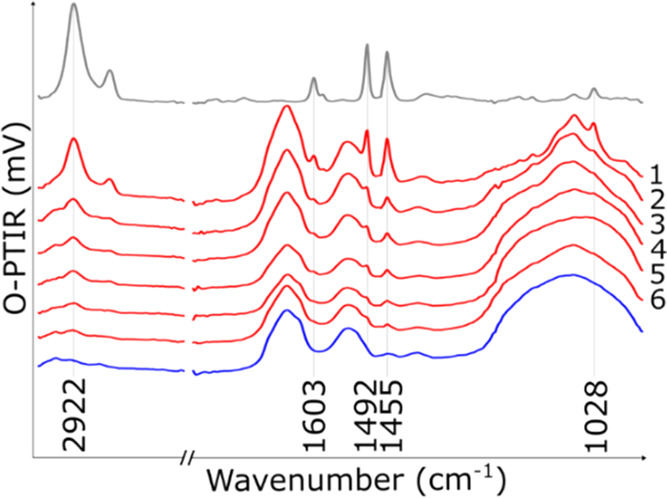
To verify correct particle detection a full O-PTIR spectrum was recorded for the individual embedded and untreated PS beads in HCT116 spheroids. The gray spectrum represents a single pristine PS bead, the blue spectrum corresponds to a sample region without PS, and the red spectra show PS beads within the spheroid (exact positions indicated in Figure 2), with clearly distinguishable PS and cell features.

The blue spectrum in Figure 3 shows a typical O-PTIR spectrum taken from a spheroid area without PS particles, while the gray spectrum was taken on an individual pristine PS bead. The red spectra were taken from PS particles within the spheroid, showing a mixed spectrum from both entities. Despite the background spectrum of the spheroid, the absorption bands of the PS beads could be distinguished unambiguously. As evident from the acquired spectral data, the ratio between the respective absorption peak amplitudes may serve as a highly selective indicator for the presence of MNPs in spatial scans and the resulting images. Although a full spectrum was acquired at each pixel, reducing the scanning process to two distinct spectral features would improve the scanning speed significantly. It took about 120 min to record the large overview image in Figure 2a at the relevant wavenumbers. The resulting image with 1883 × 1519 pixels had a size of 470.5 μm width and 379.5 μm height. Further, detailed O-PTIR measurements were conducted in selected regions to demonstrate the system imaging capabilities and chemical sensitivity of PS particles smaller than 10 μm. In the magnified view in Figure 2b the advantage of a chemically selective image compared to the chemically nonselective visual image becomes apparent. A full spectrum was recorded for each localized polymer bead, and a random spot in the surrounding cells was used as negative control for validation. The optical microscopy image displays various structures and features, which can easily be mistaken for potential MNP contamination. In contrast, the chemical image focuses on the feature of interest (PS) and is largely unaffected by structural features of the biological sample. The chemical image in Figure 2b highlights the locations of the particles in the sample, providing a ratio value for each pixel.

We further aimed toward semiautomation through binary segmentation to accelerate the data analysis process. In this binary segmentation, Class 1 represents the particle of interest, such as PS, and Class 0 encompasses everything that does not match the value of the particle of interest. This method simplifies the evaluation by categorizing each pixel as either the particle of interest or not, enhancing the speed and accuracy of the analysis, while integrating seamlessly with the transition toward semiautomated evaluations. Transitioning from manual to semiautomated evaluation processes improves efficiency and accuracy, allowing for rapid, consistent analysis while freeing up valuable human resources to focus on complex, judgment-based assessments. This shift not only streamlines routine evaluations but also ensures a more reliable and scalable approach to decision-making.

In Figure 2c we show the results of the segmentation process and their applicability for particle detection. As mentioned earlier, the images in Figure 2b are based on the calculated absorption peak ratio we used as the initial point for feeding into the segmentation algorithm. We applied a region-growing algorithm, which started from a set of seed points and expanded outward by appending nearby pixels with similar properties, as in our case ratio values, to form larger regions. This method iteratively groups pixels or subregions into regions based on predefined criteria, effectively segmenting the image into distinct areas. Finally, a postprocessing step was carried out to further refine the results. Therefore, an opening was used to remove slight noise and artifacts to fill in small holes and gaps in the segmented regions. These steps used a rectangular kernel, whose size could be adjusted, to refine the segmentation, making it cleaner and more continuous.

Based on the successful measurement of particles in the μm-regime the next iteration of our study was targeting the imaging capabilities of the O-PTIR system in the sub-μm regime (nanoplastic particles). For that purpose, 250 nm PS beads embedded in a spheroid sample were used to determine whether the detection of individual beads in that size range was possible. We observed a tendency for aggregation of this 250 nm bead size in connection with Fetal Bovine Serum (FBS), which prevented us from imaging unclustered PS beads in the spheroid. In Figure 4, one of these agglomerates within a spheroid was examined in more detail using hyperspectral imaging (HSI). Here, we recorded a complete spectrum from each individual pixel in the wavenumber range from 3000 to 2679 cm^–1^ and from 1857 to 900 cm^–1^.

**Figure 4:**
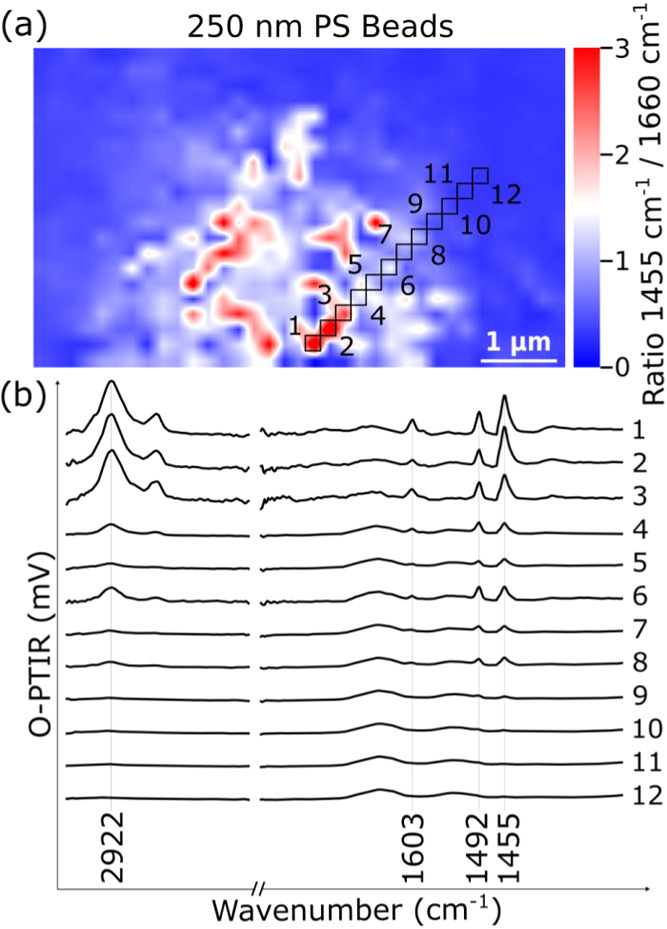
In image (a) the ratio is depicted per pixel. It is a section of agglomerated 250 nm PS beads with a total diameter of approximately 4 μm. The black squares indicate measurement points along a line from the center to the edge of the PS agglomeration. The spectra are displayed in (b) and are numbered.

The O-PTIR signal ratios of a characteristic PS band (1455 cm^–1^) and the protein-specific marker band for tissue (1660 cm^–1^) are plotted in Figure 4a. By avoiding the acquisition of full spectra and instead focusing on specific bands one could reduce acquisition times significantly. Figure 4b displays the spectral/chemical changes along the highlighted measurement positions. As one moves further from the center of the agglomerate toward the surrounding cells, the protein-specific bands become more pronounced while the PS-specific bands are getting weaker (from 1 to 12). This fading effect indicates that the agglomerate of 250 nm PS beads is found deeper within the 10 μm tissue cross-section when moving away from the center of the agglomerate.

The findings on spheroids show that chemically resolved imaging with O-PTIR is feasible and beads with diameters down to 250 nm can be localized and identified within the formed aggregates based on PS-specific absorption bands. In the next section, we present additional data where individual beads were imaged to support our claim.

### Detection of MNPs in Mouse Kidney Tissue

Building on the results obtained with spheroids, we moved to the next crucial step for actual biological and medical applications, i.e., investigations of *in* *vivo* tissue samples. O-PTIR measurements were carried out on deparaffinized FFPE mouse kidney tissue. Mouse kidney tissue was spiked post-mortem with PS beads with diameters of 10, 1, and 200 nm following the procedure described in the Materials and Methods section. Similar to the spheroids, we investigated the differently sized beads in mouse kidney tissue by focusing on the PS-specific band ratio. Consistent with our findings on the spheroid samples, the spectra of the beads with 1 μm diameter combine the spectral features of the surrounding biological material, here the mouse tissue, and the PS beads. Understandably, the contribution of the surrounding mouse tissue (5 μm thickness of tissue section) was more pronounced in the case of the smaller 200 nm beads. In the case of the 10 μm beads, however, the observed signal resembled a pure PS spectrum without any signatures of the surrounding tissue. This observation is consistent with the approximate size of the read-out laser spot, i.e., for larger particles, the surrounding tissue was not excited and thus did not contribute significantly to the O-PTIR signal.

In Figure 5, the key bands significant for PS, along with those for tissue were marked with vertical lines in the figure to highlight their relevance. Figure 6a shows an optical microscope image with an overlay of the O-PTIR false-color image based on the ratio at the two characteristic wavelengths for PS and mouse tissue. We applied the same computational procedure used in the spheroid segmentation process on the complex tissue of the mouse kidneys (Figure 6b). After implementing the region-growing algorithm, significantly more noise remained in the kidney tissue compared to the spheroid sample.

**Figure 5:**
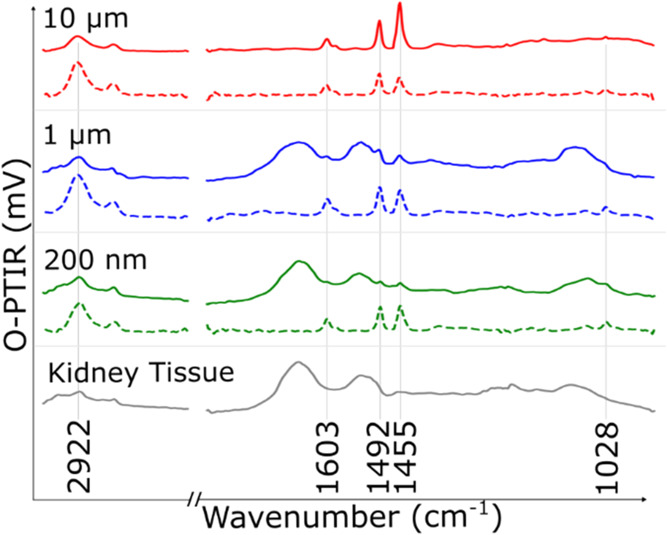
Comparison of O-PTIR signals for isolated spherical PS beads (dashed lines) with diameters of 10, 1 μm, and 200 nm compared with the signal of PS beads spiked into mouse kidney tissue (solid lines). Vertical lines indicate characteristic absorption bands of PS. The gray spectrum corresponds to kidney tissue.

**Figure 6:**
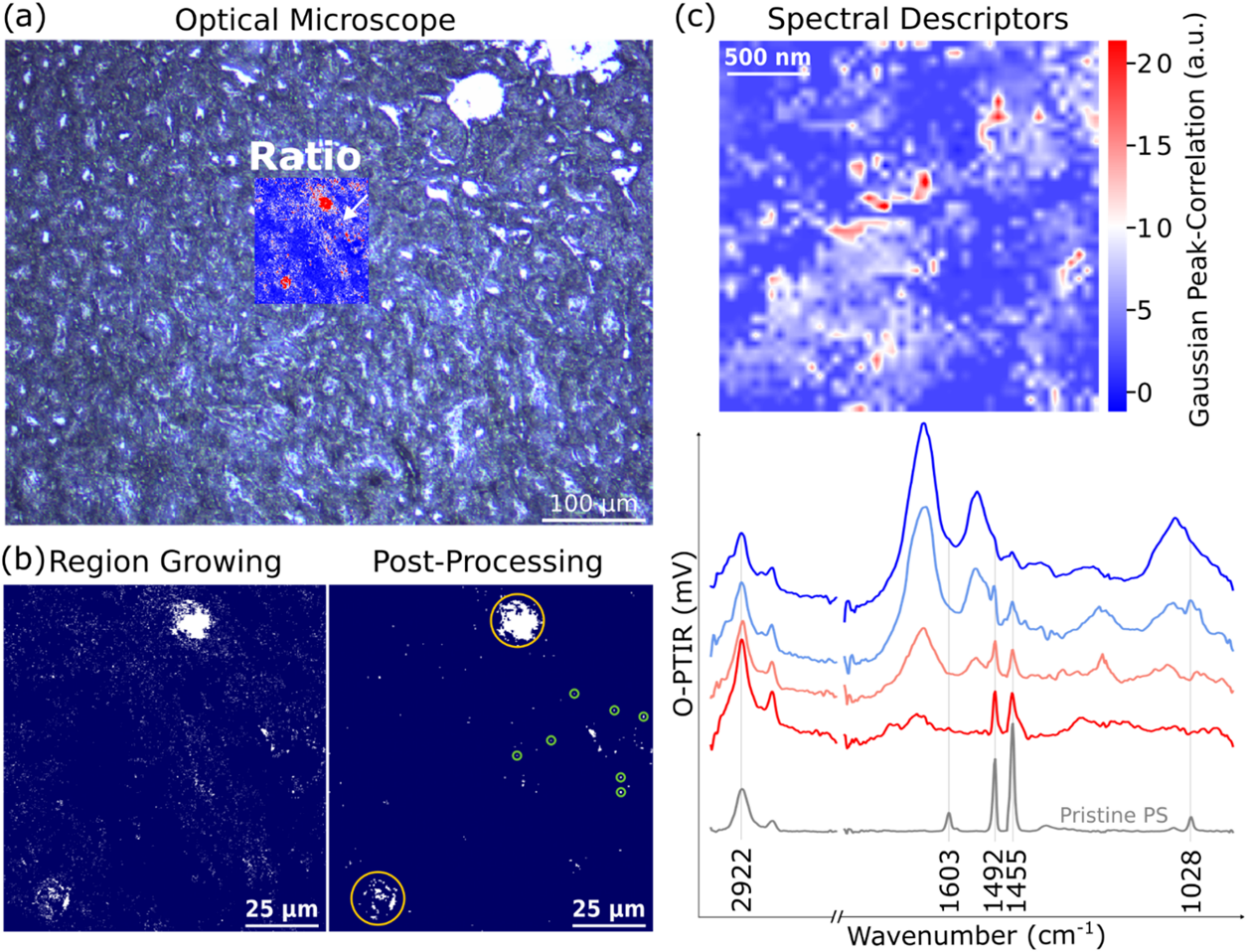
Mouse kidney tissue spiked with spherical 10, 1 μm, and 200 nm PS beads. In (a) the cross-section of a spiked mouse kidney is illustrated. The area of interest is overlapped with a ratio image. Growing segmentation and postprocessing are applied in the subimages in (b). The orange circles indicate the detected 10 μm particles, while green circles highlight some of the 1 μm particles. The white arrow in the ratio image indicates the 2.3 × 2.3 μm^2^ area recorded as an HSI, which is illustrated in (c). Therein, 200 nm particles in the mouse tissue become visible. Corresponding spectra confirming their chemical identity are shown in the bottom plot in (c).

Our postprocessing aimed to strike a balance in which particles were properly segmented without losing too much information. In Figure 6b, the 10 μm particles are marked in yellow, and some of the 1 μm particles are marked in green. The bottom left 10 μm particle is partially covered by surrounding tissue and is therefore not fully visible (confirmed by acquisition of full spectra, data not shown). While the computational procedure allowed us to filter out a significant amount of noise, some background noise remained. It could even mislabel PS particles as pristine tissue: e.g., the area where the 200 nm beads were localized was initially not recognized by the postprocessing and was falsely labeled. This suggests that the ratio method was effective for semiautomatically identifying particles of 10 and 1 μm size but did not provide enough information to clearly identify 200 nm particles in complex tissue with the employed algorithm. This can be assigned to the fact that kidney tissue is significantly more complex than spheroids. To improve reliability, more advanced segmentation algorithms are being developed to better detect particles within the tissue. Based on this finding, we recorded a high-resolution hyperspectral image (Figure 6c). For this map, we employed spectral descriptors,^25^ one centered at 1495 cm^–1^ and the other at 1455 cm^–1^. The chosen spectral descriptor calculated whether a triangle can fit under the respective absorption bands, thereby distinguishing unspecific spectral changes from those associated with actual IR bands. This method is more powerful than the ratio approach described earlier, however, it requires acquisition of actual spectra, ideally full spectra. The spectra shown at the bottom in Figure 6c correspond to the color coding of the 2D map, indicating where the influence of PS is higher (red) or lower (blue). Eventually the data obtained confirm that PS particles of all investigated sizes can be detected in mouse kidney tissue. Larger diameters were identified using the time-efficient ratio method as image contrast, while smaller particles with a 200 nm diameter required acquiring full spectral features for identification. A detailed analysis of the physical representation of the different particle sizes in the O-PTIR images is provided in the supplemental section (Figure S1).

To emphasize the implemented technique’s impressive performance, we provide a comparison of the investigated region imaged by optical microscopy (a), FTIR reflection (b), and O-PTIR (c) in Figure 7. The left picture displays the image captured with white-light microscopy in epi-configuration. Here, two beads with a diameter of 10 μm, indicated by white arrows are directly recognizable but without information about their chemical nature. Direct identification becomes more challenging with beads that are 1 μm in size, indicated by the red arrow. Although the FTIR reflection measurement suggested the presence of MNPs across the imaged area, the limited amount of light reaching the detector made it difficult to reliably identify: the spectra in the red-appearing areas were visually checked, and there were no significant PS characteristics visible with the chosen aperture size (30 × 30 μm^2^), which was the smallest possible to obtain a usable signal in reflection mode. In stark contrast to the white-light microscopy and FTIR, the O-PTIR image shows a distinct contrast between the PS beads of 10 and 1 μm size and the surroundings, also clearly revealing the shape of the particles. The spatial resolution and signal quality of the O-PTIR technique allowed an in-depth analysis of the individual particles with different sizes present in the mouse tissue, i.e., 10, 1 μm, and 200 nm.

**Figure 7:**
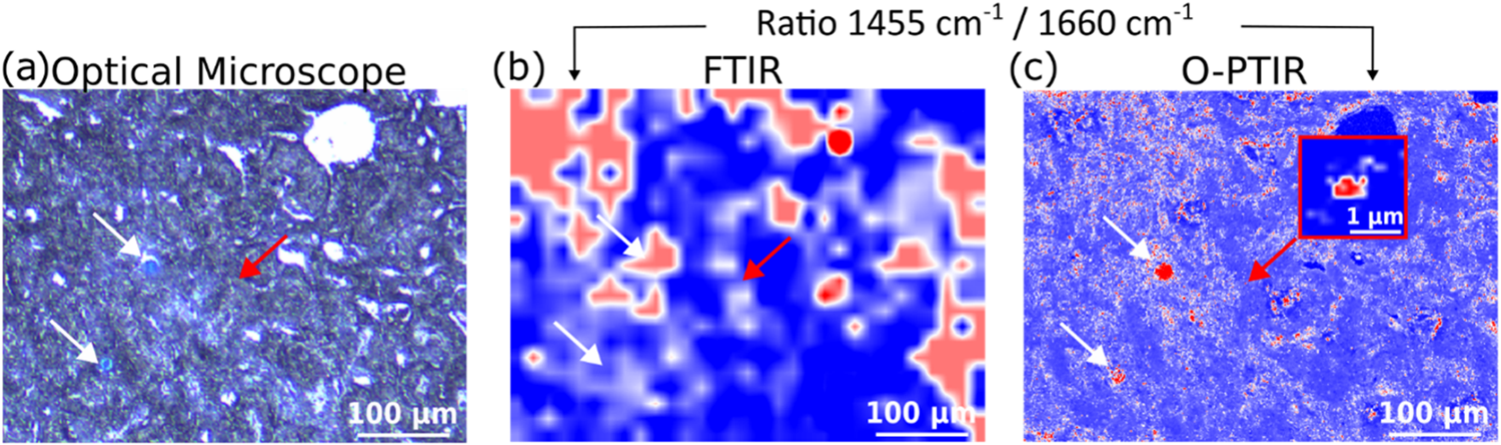
Comparison of imaging techniques in mouse kidney tissue section spiked with 10, 1 μm, and 200 nm PS-particles, the smallest particles (200 nm) are not visible in this overview image. In (a) a white-light microscopy image is shown, (b, c) show false-color images obtained with FTIR- and O-PTIR microscopy, respectively. White arrows exemplarily indicate two 10 μm particles, the red arrow indicates a 1 μm particle. Particle identity was confirmed by recording full spectra at the specific position. For the FTIR-image an aperture of 30 × 30 μm^2^ and a step width of 15 μm was used, while the O-PTIR image was taken with a step width of 250 nm.

## Conclusions

In this study, we employed O-PTIR spectroscopy to effectively localize and identify micro- and nanoplastic particles (MNPs) with diameters of 10, 1 μm, 250 and 200 nm–enabled by the significantly higher spatial resolution compared to FTIR microscopy. Artifacts, typical for infrared reflection measurements, are not observed with the O-PTIR technique, which massively improves spectra interpretation. Unlike traditional FTIR, which requires spectral acquisition at each spatial point to build a hyperspectral image, O-PTIR can capture either full hyperspectral images or images at selected wavenumbers characteristic for a distinct polymer type. This flexibility, particularly the ability to acquire data at specific wavelengths, dramatically increases image acquisition speed, while still delivering essential chemical information.

The obtained results prove the capability of O-PTIR to detect MNP particles in formalin-fixed paraffin-embedded (FFPE) tissue sections, a significant advancement over traditional methods that rely on tissue digestion and filtration. This allows for direct chemical imaging of particles within a tissue matrix, providing spatial information on their precise locations without the need for destructive sample processing. Remarkably, despite the inherent challenges in detecting particles as small as 200 nm, which are often difficult to identify even using traditional methods, O-PTIR proves capable, thereby presenting a viable alternative to FTIR.

This advancement might offer label-free and nondestructive insights into the distribution of MNPs in FFPE tissue samples, enabling the inclusion of histopathological context on particle accumulation and its potential implications. Future work will focus on refining and applying more powerful segmentation algorithms to improve detection speed and accuracy, further advancing our ability to monitor microplastic distribution in complex tissue samples.

## Supplementary Material



## Notes

Detailed examination of individual isolated polystyrene particles with 10 μm, 1 μm and 200 nm size (PDF)
