# Supplementary material for: Detection of Unlabeled Polystyrene Micro- and Nanoplastics in Mammalian Tissue by Optical Photothermal Infrared Spectroscopy
Source: Anal Chem. 2025 Aug 1;97(31):16714–22. doi: 10.1021/acs.analchem.4c05400 (PMC12356189; doi:10.1021/acs.analchem.4c05400)
Supplement: Supplementary file 1 [file ac4c05400_si_001.pdf]

Detection of Unlabeled Polystyrene Micro- and Nanoplastics in Unstained Tissue with Optical Photothermal Infrared Spectroscopy

Kristina Duswald[a,b], Verena Pichler[b,c], Verena Kopatz[b,d,e,f], Tanja Limberger[b], Verena Karl[a], David Hennerbichler[a], Robert Zimmerleiter[a], Wolfgang Wadsak[b], Mike Hettich[a], Elisabeth S. Gruber[g], Lukas Kenner[b,d,e,h,i,j *], Markus Brandstetter[a,b *]

a RECENDT GmbH – Research Center for Non-Destructive Testing, Linz, Upper Austria, 4040, Austria

b CBmed GmbH – Center for Biomarker Research in Medicine, Graz, Styria, 8010, Austria

c Department of Pharmaceutical Sciences, Division of Pharmaceutical Chemistry, University Vienna, Vienna, 1090, Austria

d Clinical Institute of Pathology, Department for Experimental and Laboratory Animal Pathology, Medical University of Vienna, Vienna, 1090, Austria

e Comprehensive Cancer Center, Medical University Vienna, Vienna, 1090, Austria

f Department for Radiation Oncology, Medical University of Vienna, Vienna, 1090, Austria

g Department of General Surgery, Medical University Vienna, Vienna, 1090, Austria

h Unit of Laboratory Animal Pathology, University of Veterinary Medicine Vienna, Vienna, 1210, Austria

i Department of Molecular Biology, Umeå University, Umeå, 90187, Sweden

j Christian Doppler Laboratory for Applied Metabolomics, Medical University Vienna, Vienna, 1090, Austria

* Co-Correspondence to MB and LK

SUPPLEMENT MATERIAL

In Figure S1 false-color chemical images of selected areas for each particle size are exemplarily shown. Individual particles were identified and confirmed by acquisition of a full spectrum. Special care was taken to locate isolated particles. The right-hand column shows the respective lateral mean intensity profiles in the x- and y-direction. We decided to use a mean spatial average for the profiles instead of a single-pixel profile to reduce noise. The image was recorded with a spacing of 100 nm, resulting in the averaging over each column or range being a factor of 10 greater than the length of the corresponding axis.

In Figure S1 a), a 10 µm PS particle is shown. Compared to spheroids, the increased complexity of the mouse kidney tissue is noticeable as an accumulation of noise around the 10 µm particle. A closer look at the obtained image reveals a particularity in detecting this bead size. The detected signal is smaller in the middle section of the particle, which results in a doughnut shape on the lateral profile (not visible in the averaged profile). We noticed a similar behavior for other particles of this size, which hinted towards an effect specific to the O-PTIR detection method related to the focusing optics and scattering effects of the mid-IR laser beam from the curved particle geometry, due to the particle size comparable to the mid-IR laser wavelength. However, closer investigation of this effect is outside of the scope of this article and will be further pursued within a separate research article. Despite this potential measurement artefact, a good agreement between the extracted particle size, measured as $1/e^2$ of the averaged spatial profiles, and the expected particle size is achieved in the case where the spot size of the optical detection was considerably smaller than the particle diameter.

In Figure S1 b) a 1 µm particle is investigated. The extracted lateral profiles show a slightly smaller size than expected. This could be due to a possible cut of the particle during microtome processing or due to tissue overlap in the border regions, i.e. the measured cross-section of the particle might appear smaller than expected.

The left part in Figure S1 c) shows the O-PTIR image of an isolated 200 nm PS particle, the smallest particle size used in our experiments. The results obtained demonstrate excellent detection capabilities for nanoplastic particles. The extracted profiles of the isolated polymer bead are shown in the right part, where one finds a $1/e^2$ of 300 nm and 330 nm for the x- and y-direction, respectively. Since the spot size of the detection laser exceeded the particle size, one would have expected to find an image size of the particle with a dimension of at least the optical $1/e^2$ spot size of 500 nm. This suggests that the final image of the particle is a convolution of the particle size and the Gaussian optical beam.

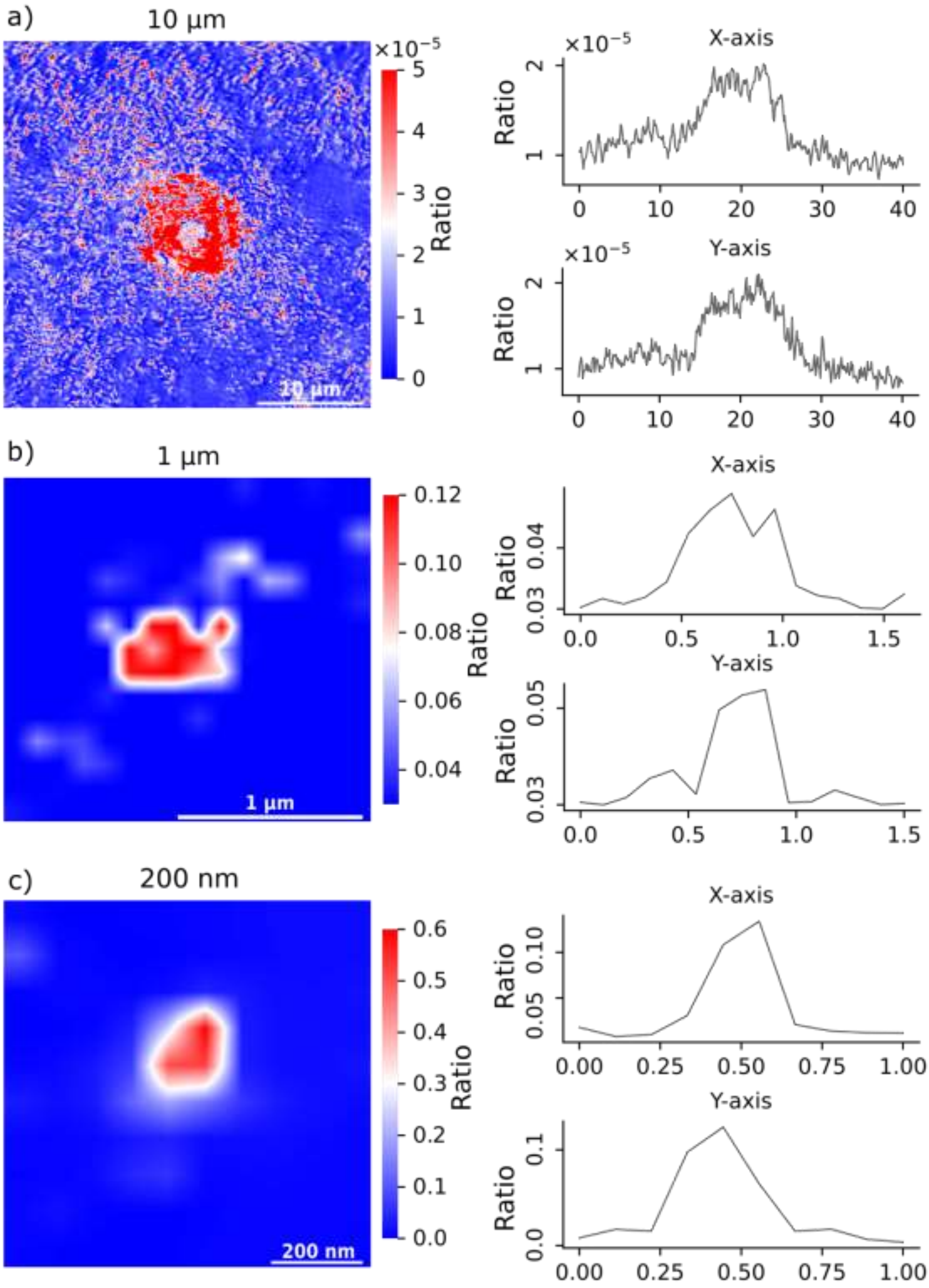

Figure S1: The left column shows the false-color image of the different particle sizes a) 10 µm, b) 1 µm, c) 200 nm) embedded in mouse tissue. The right column shows the particles' intensity profiles along the x-axis and y-axis.
